# Estimation of Human Mobility Patterns for Forecasting the Early Spread of Disease

**DOI:** 10.3390/healthcare9091224

**Published:** 2021-09-16

**Authors:** Zhengyan Li, Huichun Li, Xue Zhang, Chengli Zhao

**Affiliations:** College of Liberal Arts and Sciences, National University of Defense Technology, Changsha 410073, China; lizhengyan19@nudt.edu.cn (Z.L.); lihuichun@nudt.edu.cn (H.L.); xuezhang@nudt.edu.cn (X.Z.)

**Keywords:** human mobility, travel flow, infectious disease, COVID-19, epidemic model

## Abstract

Human mobility data are indispensable in modeling large-scale epidemics, especially in predicting the spatial spread of diseases and in evaluating spatial heterogeneity intervention strategies. However, statistical data that can accurately describe large-scale population migration are often difficult to obtain. We propose an algorithm model based on the network science approach, which estimates the travel flow data in mainland China by transforming location big data and airline operation data into network structure information. In addition, we established a simplified deterministic SEIR (Susceptible-Exposed-Infectious-Recovered)-metapopulation model to verify the effectiveness of the estimated travel flow data in the study of predicting epidemic spread. The results show that individual travel distance in mainland China is mainly within 100 km. There is far more travel between prefectures within the same province than across provinces. The epidemic spatial spread model incorporating estimated travel data accurately predicts the spread of COVID-19 in mainland China. The results suggest that there are far more travelers than usual during the Spring Festival in mainland China, and the number of travelers from Wuhan mainly determines the number of confirmed cases of COVID-19 in each prefecture.

## 1. Introduction

Human mobility has become a hot research topic in the scientific community in recent years because of its application value in many fields [1,2,3,4,5,6]. In terms of theoretical epidemiology, a large number of studies have shown that the transnational spread of many infectious diseases is closely related to individuals’ international air travel, which is considered to be the primary way of the spread of pathogens between continents [7]. Based on this, epidemiologists incorporate air travel data into epidemic spread models and have achieved satisfactory results. However, the accuracy of the prediction is limited to international spread [8,9,10,11,12,13] because international travel is dominated by air travel, and the airline operation data are easily accessible. However, there is a lack of nationwide, accurate and dynamic statistical data that describe the large-scale inter-area travel flows.

To cope with the lack of human mobility data, researchers have established spatial interaction models to estimate travel flow by using local statistical survey data. The main spatial interaction models are gravity models and radiation models [14,15], which were the main research methods used for obtaining human mobility data in the past. Huang and Mao et al. used publicly available airline operation history data to build a gravity model and estimated the number of passengers between airports around the world [16,17]. Ajelli and Balcan et al. analyzed commuting flow data from multiple countries and found a gravity model that can provide a worldwide description of commuting patterns [18,19,20]. However, spatial interaction models, such as gravity model, cannot describe the dynamic changes of human mobility in the short term, and the establishment of the model depends on the availability of historical data.

Recently, some large-size Internet companies have integrated mobile device location big data obtained from users and released a human mobility data product, opening up a new situation for estimating travel flow data [21,22,23]. At the end of 2019, a new type of coronavirus was discovered, which was later named SARS-CoV-2 [24]. The respiratory disease that the virus causes seriously threatened global public health security. Many epidemiological researchers used human mobility data products released by Internet companies to evaluate the impact of travel on the spread of COVID-19 or to build scenario models of COVID-19 [25]. We noticed that most of the data used in these studies are relative index data (non-actual number of travelers) [26,27,28], and some data are travel flows calculated based on the ratio of the number of mobile devices to the permanent census population in the area [29,30,31,32,33]. However, the reality is that the actual population stock of an area contains a large number of mobile people, which is quite different from the permanent population and is very difficult to calculate. As a result, the travel flows calculated by using mobile devices and census population data may not be sufficiently accurate.

In this study, we propose an algorithm model that combines mobile device location big data with real airline operation data to estimate the dynamic travel flows because it is difficult to collect the number of inter-prefecture travelers. In addition, we established a simplified deterministic SEIR-metapopulation model based on the early spread of COVID-19 in mainland China to demonstrate the use of estimated travel flows.

## 2. Materials and Methods

### 2.1. Data

The original data used in the study were obtained from the migration big data platform developed by Baidu and Tencent. Baidu and Tencent are the two largest Internet companies in China and have more than 500 million active users, covering almost all mobile phone users in the country. They provide location services in their applications, and the collected location big data can fully and truly reflect the status of human mobility. The Baidu Map Migration Big Data Platform calculates and processes hundreds of billions of positioning data collected every day and releases the migration proportion data from the provincial and prefecture levels in mainland China [34]. A detailed description of China’s administrative divisions is described in Appendix A. Tencent location big data analyzes massive user location data to calculate the proportion of different transportation modes on each arrival/departure route between all cities [35]. In this study, we obtained the migration proportion data from 1 January 2020 to 31 January 2020 from the Baidu Map Migration Big Data Platform, covering 337 administrative regions, including 333 prefectures and four municipalities. The proportion of different transportation modes on each of the 10 routes arriving and departing from Beijing was obtained from the Tencent location big data platform.

The daily airline operation data of all civil airports in mainland China were obtained from VariFlight Company [36], including airport information, the three-character codes of departure and arrival airports, and the actual number of passengers on each route (only some of the data are the number of seats).

In order to reconstruct the spread of COVID-19 in mainland China using a mathematical model, we obtained COVID-19-related data from the National Health Commission of China [37], including the number of daily cumulative confirmed cases of all prefectures from 24 January 2020 to 16 February 2020.

All the data mentioned above are anonymous aggregated data and do not involve personal information.

### 2.2. Estimating Human Mobility Patterns in Mainland China

#### 2.2.1. Overview of the Methodology

**Figure 1:**
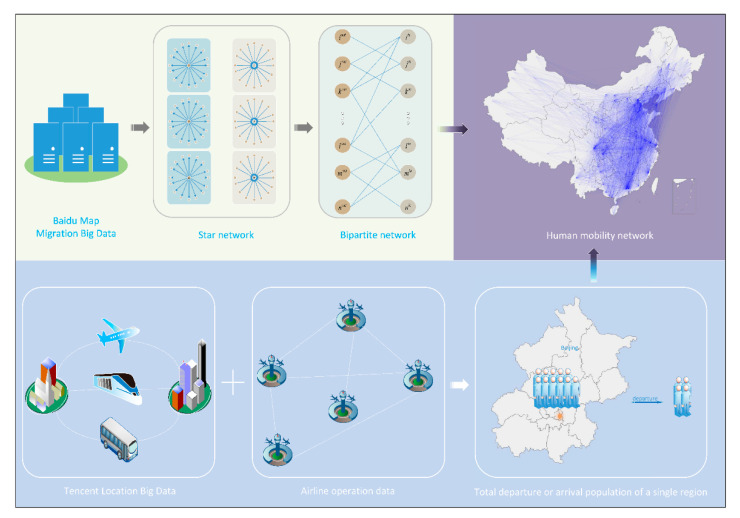
Overview of the data and model framework.

In order to estimate the human mobility patterns in mainland China and build a human mobility network (directed network) between all prefectures, we propose a data fusion algorithm model based on the network science approach, which can estimate travel flow data in mainland China. Here, we use the network adjacency matrix $A=\left(A_{ij}\right)$ to describe the travel flow, and the matrix element $A_{ij}$ represents the estimated number of travelers from prefecture i to the other prefecture j. Figure 1 gives an overview of the data and algorithm steps of the modeling framework for estimating the human mobility network.

The modeling is mainly carried out in three steps. First, the relationship between the daily total departure/arrival population of different prefectures is obtained from the migration proportion data and converted into a bipartite network. Here, the total departure population of a prefecture indicates the number of all individuals leaving this prefecture on one day, and the total arrival population of a prefecture indicates the number of all individuals entering this prefecture on one day. This bipartite network is undirected and weighted and we can estimate the total departure/arrival population of all prefectures by using it if the total departure/arrival population of an arbitrary prefecture is known. Second, the total departure/arrival population of an arbitrary prefecture (such as the total departure population of Beijing) is estimated using the ratio estimation method combined with airline operation data and the proportions of transportation modes. Lastly, using the breadth-first traversal algorithm, the total departure/arrival population of all prefectures is estimated. Furthermore, the travel flows between any two prefectures are estimated. More details about the model algorithm are described in Section 2.2.2.

#### 2.2.2. Model

**Figure A1:**
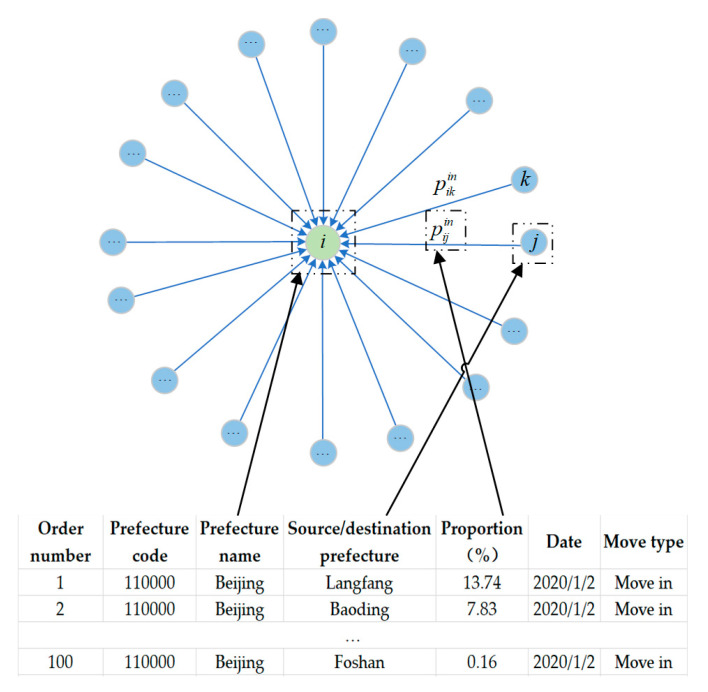
Schematic diagram of converting the migration proportion data of prefecture into a star network.

Step 1: Generate star structure network. All prefectures are coded and sorted, and the migration proportion data of each prefecture are sequentially converted into a star network of arrival type and a star network of departure type. Specifically, the migration proportion data of a prefecture list 100 sources, 100 destinations, and the proportion of people on each route out of the total people entering (or leaving) the prefecture. A detailed description of the migration proportion data is available in Appendix B. For each prefecture, the central node of the star network of arrival type (or departure type) corresponds to the prefecture, and the nodes connected only to the central node correspond to the sources (destinations) listed in the migration proportion data. The weight of the edge in the network is the migration proportion. The schematic diagram of converting the migration proportion data of prefecture into a star network is shown in Figure A1.

**Figure 2:**
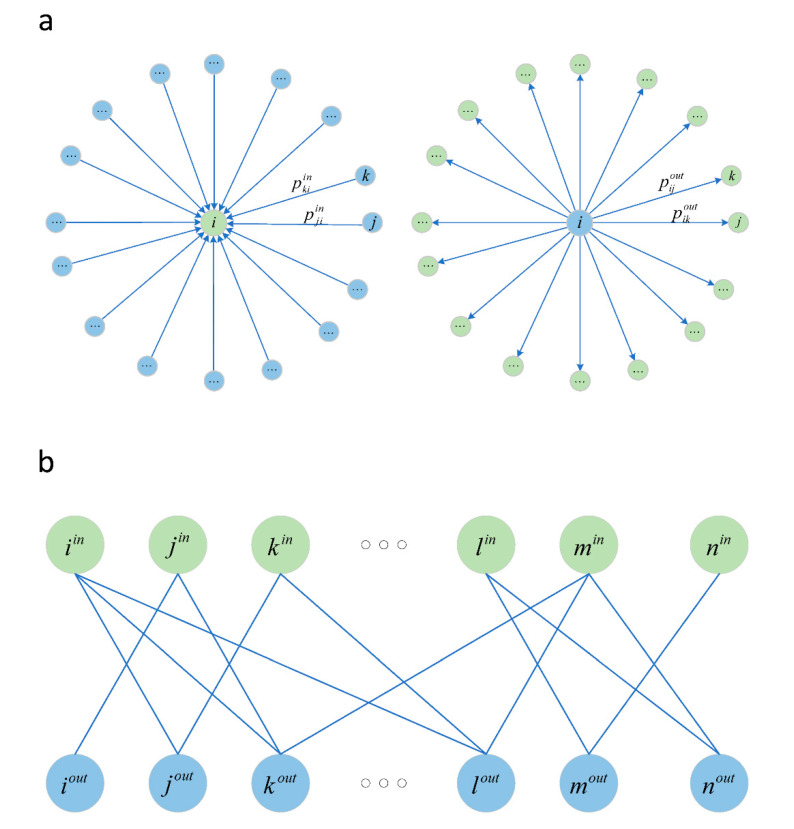
Network structure transformed from migration proportion data. (**a**) A star network; (**b**) a two-part network.

Here, the star network of arrival type can be expressed as $G_{i}^{in}\left(V_{i}^{in},E_{i}^{in}\right)$, where $V_{i}^{in}$ represents the node set of the star network, including the central node i and its 100 neighbor nodes; $E_{i}^{in}$ represents the edge set of the star-structure network, including 100 weighted directed edges pointing to the central node i, and the weight of $e_{ji}^{in}$, the edge connected from j to i, is set to $p_{ji}^{in}$. Similarly, the star network of departure type can be expressed as $G_{i}^{out}\left(V_{i}^{out},E_{i}^{out}\right)$. Figure 2a shows a schematic diagram of a star network of arrival type and a star network of departure type.

Step 2: Generate a bipartite network. For any two prefectures i and j, the travel flow from prefecture i to prefecture j is set to $n_{ij}$, the number of total people leaving the prefecture i is set to $N_{i}^{out}$, and the number of total people entering the prefecture j is set to $N_{j}^{in}$. Obviously, there is the following conservation relationship between the number of migrants:

(1) $$p_{ij}^{out}\cdot N_{i}^{out}=n_{ij}=p_{ij}^{in}\cdot N_{j}^{in},$$

Under the premise that there are data on the migration proportions $p_{ij}^{out}$ and $p_{ji}^{in}$, if $N_{i}^{out}$ is known, $N_{j}^{in}$ can be calculated according to the equation. Based on the conservation relationship (Equation (1)), we hope to estimate the total arrival and total departure populations of each prefecture from the total arrival (or departure) population of an arbitrary prefecture through multiple iterations.

In the model, we implemented this iterative estimation process by traversing the bipartite network. First, in order to build a bipartite network, we generated a node of arrival type and a node of departure type in the bipartite network that correspond to each prefecture. For example, corresponding to prefecture i, $i^{in}$ represents the node of arrival type and $i^{out}$ represents the node of departure type. In the bipartite network, if there is the edge $e_{i^{out}j^{in}}$ between $i^{out}$ and $j^{in}$, we can calculate $N_{i}^{out}$ from $N_{j}^{in}$, or calculate $N_{j}^{in}$ from $N_{i}^{out}$. Whether there is an edge between $i^{out}$ and $j^{in}$ can be inferred from the structural information of the star networks. Specifically, if the node j is in the star network $G_{i}^{out}$, and the node i is in the star network $G_{j}^{in}$, nodes $i^{out}$ and $j^{in}$ are connected by the edge $e_{i^{out}j^{in}}$ in the bipartite network, and the weight of the edge is set to $p_{ij}^{out}∕p_{ij}^{in}$. After traversing all the nodes, we built the bipartite network $G=\left(V_{in},V_{out},E\right)$, where $V_{in}=\left\{i^{in},j^{in},\cdots \right\}$ represents the set of all arrival type nodes, and $V_{out}=\left\{i^{out},j^{out},\cdots \right\}$ represents the set of all departure type nodes.

Step 3: Estimate the total departure/arrival population of all prefectures. To estimate the total departure/arrival population of all prefectures, we need to input the state value of an arbitrary node of the bipartite network G, that is, the total arrival or total departure population of the prefecture corresponding to the node. Assume that total the departure/arrival population of prefecture i is input as the initial information. According to the airline operation data, we can obtain the number of air passengers $n_{ij}^{air}$ from prefecture i to prefecture j. Combining the proportion of air passengers $p_{ij}^{air}$ to all travelers from prefecture i to prefecture j, we can estimate the number of travelers from the prefecture i to the prefecture j:

(2) $$n_{ij}=n_{ij}^{air}/p_{ij}^{air},$$

According to the estimation method introduced above, the number of travelers on multiple departure routes of prefecture i can be estimated. Since the estimated number of travelers $n_{ij}$ on each route is proportional to migration proportion $p_{ij}^{out}$,

(3) $$n_{ij}=N_{i}^{out}\cdot p_{ij}^{out},$$

Furthermore, the ratio estimation method is used for estimating the total departure population of prefecture i, namely

(4) $${\hat{N}}_{i}^{out}=\frac{\bar{n_{ij}}}{\bar{p_{ij}^{out}}}=\frac{\sum_{j}n_{ij}}{\sum_{j}p_{ij}^{out}},$$

After estimating the total departure population of prefecture i, the breadth-first traversal algorithm is applied to the bipartite network G to traverse all nodes to estimate the total arrival and total departure populations of all prefectures.

Step 4: Build the human mobility network. In this study, we describe the human mobility pattern in mainland China in the form of a weighted directed network. The nodes of the human mobility network are all prefectures in mainland China. Edges in the network characterize the state of travel between the prefectures. Specifically, the matrix element $A_{ij}$ of the network adjacency matrix A represents the estimated number of travelers from prefecture i to the other prefecture j.

Here, we estimate the travel flows between prefectures using the estimation results of the total departure/arrival population of all prefectures and the migration proportion data. First, we use the estimation results of the total departure population of all prefectures. For example, the estimated total departure population of prefecture i is ${\hat{N}}_{i}^{out}$. For all neighbor nodes of i in the departure type star network $G_{i}^{out}$, such as the node j, the travel flow from prefecture i to prefecture j is estimated to be ${\hat{N}}_{i}^{out}\cdot p_{ij}^{out}$. Then, we use the estimation results of the total arrival population of all prefectures. For example, the estimated total arrival population of prefecture i is ${\hat{N}}_{i}^{in}$. For all neighbor nodes of i in the arrival type star network $G_{i}^{in}$, such as the node j, if the travel flow from prefecture j to prefecture i has not been estimated, the flow is estimated to be ${\hat{N}}_{i}^{in}\cdot p_{ji}^{in}$. For routes for which the travel flow cannot be estimated, the travel flow on the route is set to 0. The above is the method for building a human mobility network. The pseudocode of the algorithm used for generating the bipartite network and estimating the total arrival and total departure population of each prefecture is shown in the Supplementary Material.

### 2.3. Modeling the Spread of Epidemics Using Human Mobility Data

The outbreak of COVID-19 in Wuhan coincided with the Spring Festival travel season in China, and a large number of returnees left or passed through Wuhan. After clarifying the infectiousness of COVID-19, the Chinese government adopted strict intervention strategies, including locking down Wuhan and restricting travel across mainland China. In order to verify the effectiveness of the travel flows estimated by our model in predicting epidemics, understanding the early propagation dynamics of COVID-19, and evaluating the effectiveness of intervention strategies, we established a simplified spatial mechanism model of COVID-19 to simulate its spread in mainland China.

**Figure A2:**
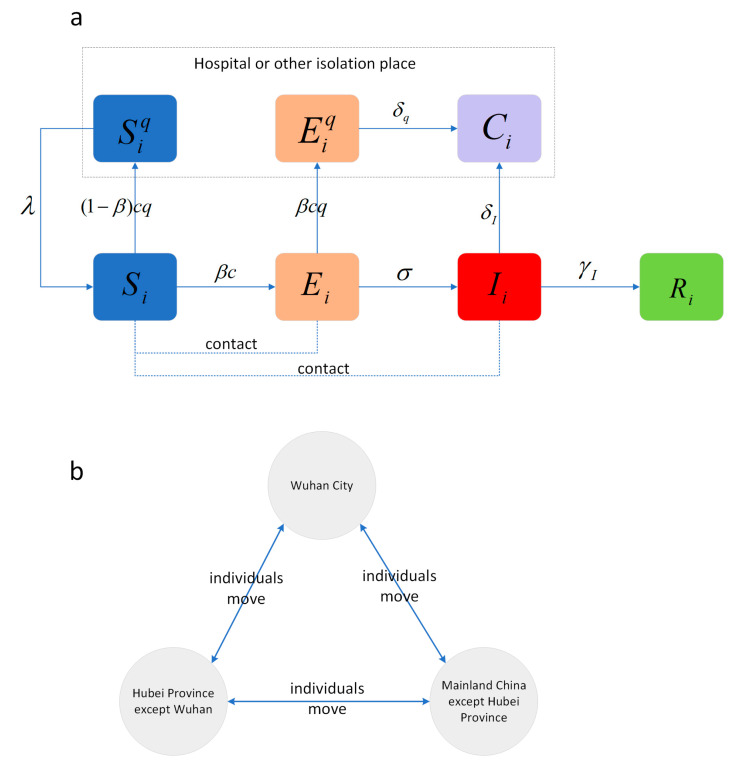
Schematic diagram of epidemic simulation model. (**a**) Schematic diagram of compartment model; (**b**) schematic diagram of a mobility network composed of three subpopulations.

Based on the traditional SEIR compartment model we established a deterministic SEIR-metapopulation model that incorporates human mobility factors and considers intervention strategies such as quarantine and travel restrictions [38]. In detail, considering the complexity of establishing a stochastic SEIR model on 337 prefectures, we divided mainland China into 3 subpopulations, namely Wuhan City, Hubei Province (excluding Wuhan), and mainland China (excluding Hubei Province). The estimated travel flows between 337 prefectures were integrated into the travel flows between the three subpopulations. The corresponding human mobility network is shown in Figure A2b. Individuals within subpopulation i are divided into various compartments according to the infection and isolation status, namely $S_{i}$ (i.e., susceptible individuals who are not isolated), $E_{i}$ (i.e., infected individuals who are during the incubation period and not isolated), $I_{i}$ (i.e., infected individuals who are symptomatic and not isolated), $S_{i}^{q}$ (i.e., susceptible individuals who are isolated), $E_{i}^{q}$ (i.e., infected individuals who are during the incubation period and are isolated), and $C_{i}$ (infected individuals who were diagnosed at hospital and isolated).

In the metapopulation model, travel flows on different dates are considered to be independent of each other, that is, in each time step, the movement of the individuals in the previous time step is not considered. The quantity of state of each compartment in each subpopulation is updated according to the human mobility network adjacency matrix $M={\left(m_{ij}\right)}_{3\times 3}$, where $m_{ij}$ represents the travel flow from subpopulation i to subpopulation j in a unit of time. The gist of the above assumptions is that we do not mark individuals according to their original subpopulations (e.g., homes in the framework considering commuting patterns), and at each time step, the same travel probability applies to all individuals in the subpopulation without having to remember their source.

Considering that some infected persons with obvious symptoms cannot participate in travel normally, the proportion of symptomatic infected individuals that can travel normally is assumed to be $k_{I}\left(k_{I}<1\right)$ in the model. In addition, isolated individuals cannot travel between subpopulations. $N_{i}^{m}\left(t\right)$ represents the number of individuals in the subpopulation i that can travel between subpopulations. At the start of each simulated day, travelers move to their destinations via the human mobility network, and the travel process is represented by the following difference equations:

(5) $$\{\begin{array}{l} N_{i}^{m}\left(t\right)=S_{i}\left(t\right)+E_{i}\left(t\right)+k_{I}I_{i}\left(t\right)+R_{i}\left(t\right)\\ \Delta S_{i}\left(t\right)=\overset{N}{\underset{j=1}{\sum }}\frac{S_{j}\left(t\right)m_{ji}\left(t\right)}{N_{j}^{m}\left(t\right)}-\frac{S_{i}\left(t\right)\sum_{j=1}^{N}m_{ij}\left(t\right)}{N_{i}^{m}\left(t\right)}\\ \Delta E_{i}\left(t\right)=\overset{N}{\underset{j=1}{\sum }}\frac{E_{j}\left(t\right)m_{ji}\left(t\right)}{N_{j}^{m}\left(t\right)}-\frac{E_{i}\left(t\right)\sum_{j=1}^{N}m_{ij}\left(t\right)}{N_{i}^{m}\left(t\right)}\\ \Delta I_{i}\left(t\right)=k_{I}\left(\overset{N}{\underset{j=1}{\sum }}\frac{I_{j}\left(t\right)m_{ji}\left(t\right)}{N_{j}^{m}\left(t\right)}-\frac{I_{i}\left(t\right)\sum_{j=1}^{N}m_{ij}\left(t\right)}{N_{i}^{m}\left(t\right)}\right)\\ \Delta R_{i}\left(t\right)=\overset{N}{\underset{j=1}{\sum }}\frac{R_{j}\left(t\right)m_{ji}\left(t\right)}{N_{j}^{m}\left(t\right)}-\frac{R_{i}\left(t\right)\sum_{j=1}^{N}m_{ij}\left(t\right)}{N_{i}^{m}\left(t\right)} \end{array}$$

After updating the individual movements of all subpopulations, the transfer of individuals between different compartments in each subpopulation based on the epidemiological natural history of COVID-19 and the implementation of intervention strategies is modeled by the following:

(6) $$\{\begin{array}{l} N_{i}\left(t\right)=S_{i}\left(t\right)+E_{i}\left(t\right)+I_{i}\left(t\right)+R_{i}\left(t\right)\\ \Delta S_{i}\left(t\right)=-\left(\beta c\left(t\right)+\left(1-\beta \right)c\left(t\right)q\left(t\right)\right)\frac{S_{i}\left(t\right)}{N_{i}\left(t\right)}\left(I_{i}\left(t\right)+\upsilon E_{i}\left(t\right)\right)+\lambda S_{i}^{q}\left(t\right)\\ \Delta E_{i}\left(t\right)=\beta c\left(t\right)\left(1-q\left(t\right)\right)\frac{S_{i}\left(t\right)}{N_{i}\left(t\right)}\left(I_{i}\left(t\right)+\upsilon E_{i}\left(t\right)\right)-\sigma E_{i}\left(t\right)\\ \Delta I_{i}\left(t\right)=\sigma E_{i}\left(t\right)-\left(\delta_{I}\left(t\right)+\gamma_{I}\right)I_{i}\left(t\right)\\ \Delta S_{i}^{q}\left(t\right)=\left(1-\beta \right)c\left(t\right)q\left(t\right)\frac{S_{i}\left(t\right)}{N_{i}\left(t\right)}\left(I_{i}\left(t\right)+\upsilon E_{i}\left(t\right)\right)-\lambda S_{i}^{q}\left(t\right)\\ \Delta E_{i}^{q}\left(t\right)=\beta c\left(t\right)q\left(t\right)\frac{S_{i}\left(t\right)}{N_{i}\left(t\right)}\left(I_{i}\left(t\right)+\upsilon E_{i}\left(t\right)\right)-\delta_{q}\left(t\right)E_{i}^{q}\left(t\right)\\ \Delta C_{i}\left(t\right)=\delta_{I}I_{i}\left(t\right)+\delta_{q}E_{i}^{q}\left(t\right) \end{array}$$

Medical researches show that individuals with no symptoms (during the incubation period) infect others just like the symptomatic [39,40,41]. Thus, in this model, new infections are mainly transformed from susceptible individuals who had contact with infected individuals who have not been isolated ($I_{i}$ and $E_{i}$). In order to make the model as realistic as possible, while avoiding making the model too complicated, we set several auxiliary parameters. For instance, c(t) represents the average number of effective contacts between $I_{i}$ and $S_{i}$ in a day. Similarly, c(t)·υ represents the average number of effective contacts between $E_{i}$ and $S_{i}$ in a day. β represents the infection probability of each effective contact. A proportion of close contacts are quarantined (isolated) due to contact tracking, and the proportion is set to q(t). If the individuals are isolated during the incubation period, they will be classified into the $E_{i}^{q}$ compartment; otherwise (if they had close contact with an infectious individual but have not been infected), they will be classified into the $S_{i}^{q}$ compartment. λ represents the rate of release from isolation. In other words, 1/λ is the duration of isolation in $S_{i}^{q}$. Infected individuals who have not been quarantined (i.e., $I_{i}$) are diagnosed at a rate of $\delta_{I}$ every day. According to the “Protocol on Prevention and Control of COVID-19 (Edition 6)” issued by the National Health Commission of China [42], for individuals isolated due to close contact tracing, their respiratory specimens or serum will be detected as soon as they are isolated. This means that most of isolated infected people will be detected positive for novel coronavirus nucleic acid or IgM in serum before they have symptoms such as fever. These persons are called “asymptomatic infected persons who have been discovered” in China. They will be diagnosed as a confirmed case as soon as obvious symptoms appear on them. Correspondingly, we assume that infected individuals who are during the incubation period and isolated (i.e.,$E_{i}^{q}$) are diagnosed at a rate of $\delta_{q}$. σ represents the transformation rate from $E_{i}$ to $I_{i}$. $\gamma_{I}$ represents the rate of recovery of infected individuals who have not been quarantined (i.e., $I_{i}$). The schematic diagram of the SEIR compartment model is shown in Figure A2a.

We simulated the spread of COVID-19 from 1 January 2020 and the simulation was divided into two periods. The first period was from 1 January to 23 January (Wuhan was in lockdown from 23 January), in which period, the estimated travel flow data were used for modeling the spatial propagation of COVID-19 in mainland China. In order to simplify the model, we set some parameters to be constants in the two periods. The average number of daily effective contacts of individuals was set to $c\left(t\right)=c_{1}$, the diagnosis rate of symptomatic infected individuals ($I_{i}$) was set to $\delta_{I}\left(t\right)=\delta_{I1}$, and the diagnosis rate of quarantined exposed individuals ($E_{i}^{q}$) was set to $\delta_{q}\left(t\right)=\delta_{q1}$. The second period was from 24 January to 31 March. Due to strict quarantine and travel restrictions, in this period, the travel between Wuhan and other prefectures of mainland China were cut off. Accordingly, the average number of daily effective contacts of individuals was set to $c\left(t\right)=c_{2}$, the diagnosis rate of symptomatic infected individuals was set to $\delta_{I}\left(t\right)=\delta_{I2}$, and the diagnosis rate of quarantined exposed individuals was set to $\delta_{q}\left(t\right)=\delta_{q2}$.

According to related research on social contact patterns, the average number of social contacts in China has decreased significantly after the Chinese government clarifying the infectiousness of COVID-19 [28,43]. Thus, we assumed that $c_{2}$ was less than $c_{1}$. In addition, with the extensive use of testing reagents, the rate at which infected persons were tested and diagnosed was also significantly faster in the second period. Accordingly, we set that $\delta_{I1}$ was less than $\delta_{I2}$ and $\delta_{q1}$ was less than $\delta_{q2}$. Since the incubation period of most infected individuals will not exceed 14 days, the isolation policy in China was that quarantined individuals will be released if they are not diagnosed with the virus within 14 days. Therefore, λ was set to 1/14.

Some parameters in the model were unknown, and the set of unknown parameters was denoted as $\Theta \;=\left\{\beta ,c_{1},c_{2},q,\upsilon ,\sigma ,\gamma_{I},\delta_{I1},\delta_{I2},\delta_{q1},\delta_{q2}\;\right\}$. In order to simulate the early spread of covid-19 in China as realistically as possible, we hope to obtain a set of parameters so that the error ($\overset{N}{\underset{t=0}{\sum }}{\left|C\left(t\right)-\hat{C}\left(t\right)\right|}^{2}$) between the simulated number of confirmed cases and the real number of confirmed cases is as small as possible. Just as $f=\overset{N}{\underset{t=0}{\sum }}{\left|C\left(t\right)-\hat{C}\left(t\right)\right|}^{2}$ is nonlinear and the parameters are constrained, this kind of problem of finding the global minimizer of f is called the constrained nonlinear programming problem (CNLP) [44]. Thus, the parameter estimation problem of the model can be expressed as the following constrained nonlinear optimization problem:

(7) $$P_{0}:\underset{\Theta }{\min}\overset{N}{\underset{t=0}{\sum }}{\left|C\left(t\right)-\hat{C}\left(t\right)\right|}^{2}s.t.\{\begin{array}{l} c_{1}>c_{2}\\ \delta_{I1}<\delta_{I2}\\ \delta_{q1}<\delta_{q2}\\ \Theta_{U}\geq \Theta \geq \Theta_{L} \end{array}$$

## 3. Results

**Figure 3:**
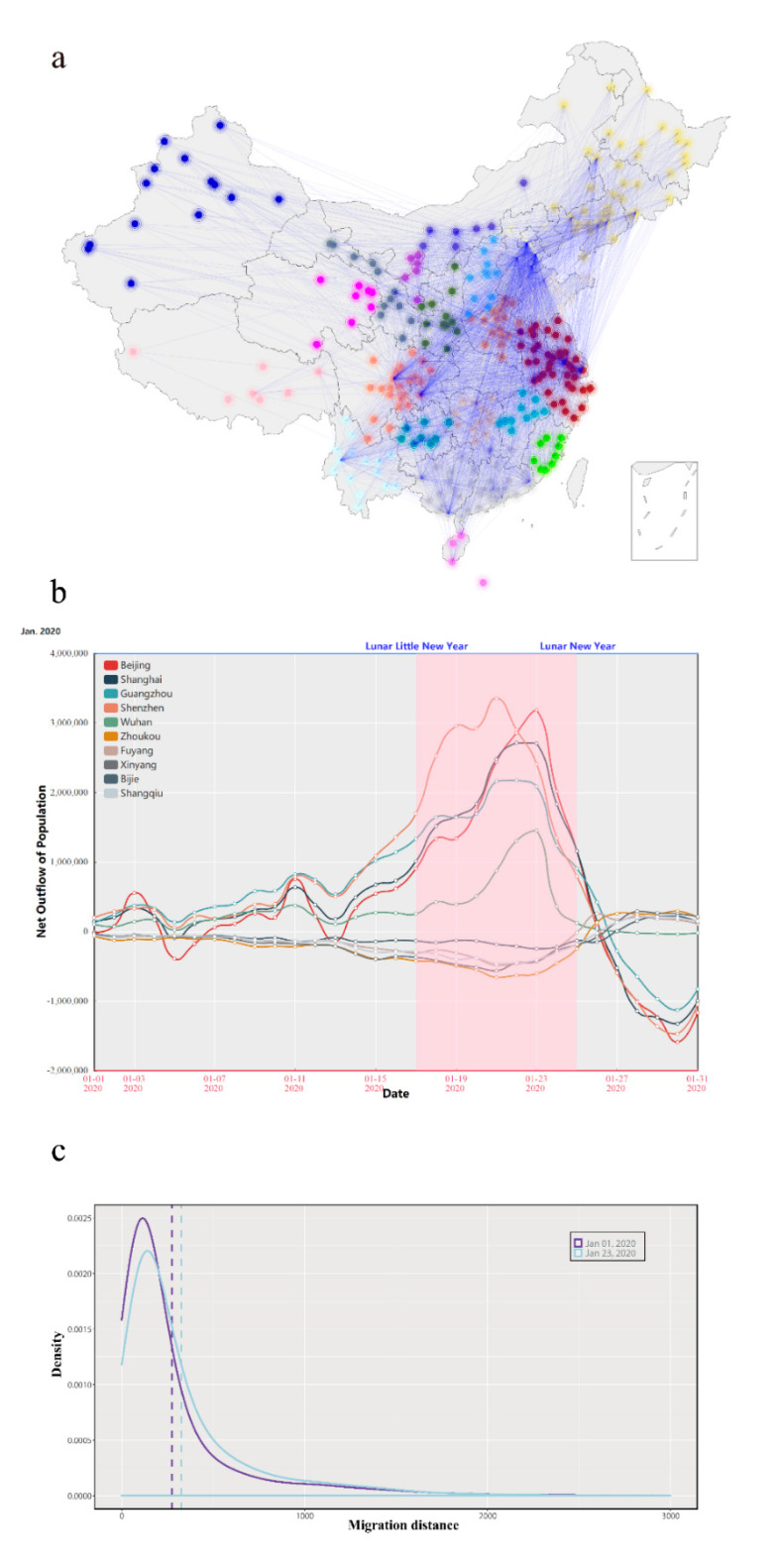
Estimated human mobility pattern in mainland China. (**a**) Human mobility network with community division; (**b**) variation curve of the number of net outflows (the departure population minus the arrival population) in 10 cities in January 2020; (**c**) probability density distribution curve of individual travel distances.

In order to accurately understand the human mobility patterns in mainland China, we designed an inter-prefecture travel flow estimation model based on mobile device location big data and airline operation data. Figure 3 shows the estimated inter-prefecture human travel patterns in mainland China using the model. Figure 3a shows the human mobility network of prefectures in mainland China during the Spring Festival. In order to show the characteristics of travel in China, we used the infomap algorithm proposed by Rosvall and Bergstrom to perform a simple community division on this human mobility network [45]. The infomap algorithm is a method of identifying community structure in directed and weighted networks (especially networks inherently characterized by flows). In the picture, all prefectures are divided into 21 communities, and nodes with the same color belong to the same division community. Nodes assigned to the same network community indicates that the communication between these nodes is more frequent and closer than those belonging to different communities. In the map, the areas separated by gray dotted lines are different provinces. It is obvious that prefectures belonging to the same province are usually divided into the same network community, indicating that Chinese people are more inclined to travel to prefectures in the province in which they were born.

Figure 3b depicts the change in the number of net outflows (the total departure population minus the total arrival population) of 10 cities in January 2020. As the matrix element $A_{ij}$ represents the estimated number of travelers from city i to another city j in one day, the net outflow of city i is equal to $\overset{N}{\underset{j=1}{\sum }}A_{ij}-\overset{N}{\underset{j=1}{\sum }}A_{ji}$. We selected the top five cities for the net outflow population and the top five cities for the net inflow (the opposite of net outflow) population. Each line represents a city. It can be clearly seen from the figure that during the Spring Festival travel season, large cities, such as Beijing and Shanghai, are dominated by population outflows (the total departure population is greater than the total arrival population), especially during the peak period (Chinese Little New Year to Chinese New Year), during which millions of people leave every day. In China, there will be a large number of people going to work or study in big cities. The Spring Festival is the most important festival in China. Before the Spring Festival, these people will leave the big cities and return to their hometowns to celebrate the Spring Festival. Especially in the days leading up to the Spring Festival, there will be a very large number of people returning hometown. We guess that this pattern of human mobility will lead to the result that it is easier for the epidemics that occurred in big cities during the Spring Festival to spread to small cities than usual. After the Spring Festival, the situation reversed and people began to return to these big cities to work. However, due to travel restrictions, the scale was significantly smaller than during the Spring Festival. Figure 3c shows the density distribution of individual travel distances. Obviously, the human mobility patterns are dominated by short- and medium-distance trips, and the vast majority of travel takes place within 100 km. In addition, the average travel distance during the Spring Festival travel season (blue curve) is slightly larger than that of daily travel (purple curve), which may be because, during the Spring Festival, more migrant workers return to remote hometowns.

**Figure 4:**
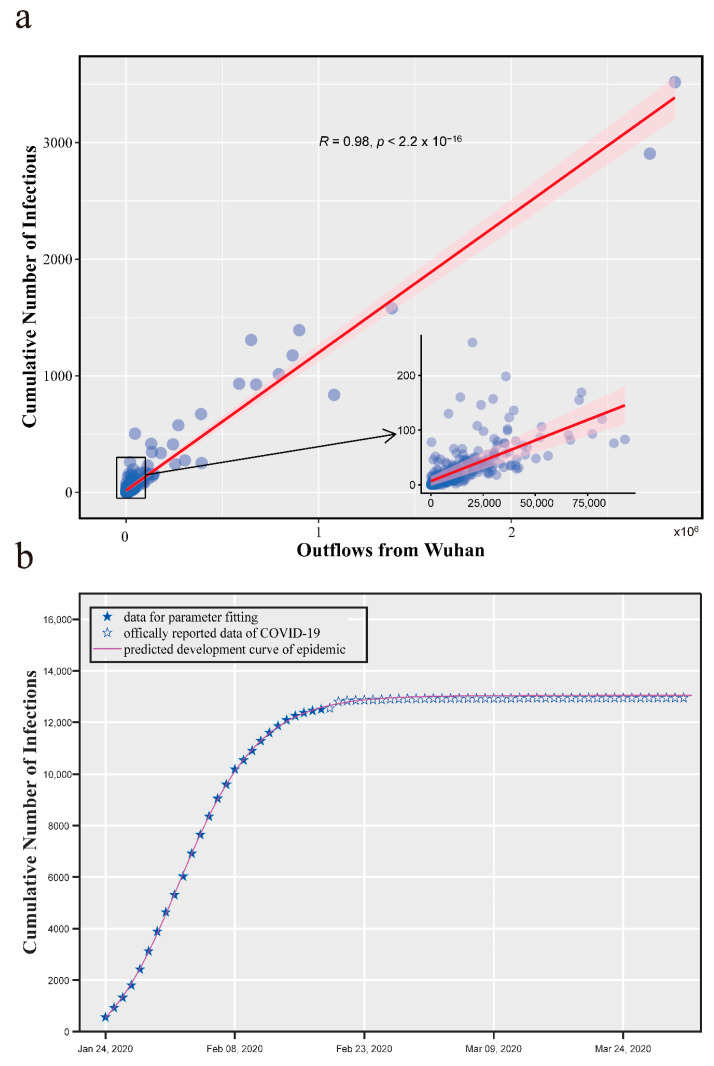
Human mobility and COVID-19. (**a**) Correlation analysis of the cumulative number of confirmed cases and the number of travelers from Wuhan; (**b**) development curve of COVID-19 predicted by the model.

In order to verify the effectiveness of the travel flow data estimated by our model in predicting epidemics, first, we conducted a correlation analysis on the cumulative number of confirmed cases of COVID-19 and the number of travelers from Wuhan. Figure 4a shows that the cumulative number of confirmed cases in each prefecture is highly correlated with the number of travelers from Wuhan, with a Pearson correlation coefficient value of 0.98, and a significance level of $P<2.2\times 10^{-16}$. This is consistent with the conclusion that the spatial transmission of epidemics is mainly affected by human mobility.

Furthermore, we established a deterministic SEIR-metapopulation model that reproduces the spread of COVID-19 in mainland China to demonstrate the significance of the estimated travel flow data for the spatial spread mechanism model of epidemics. In the early stage of the epidemic, the health department had insufficient knowledge of the new virus and a lack of diagnostic programs, which resulted in a large difference between the number of reported confirmed cases and the actual number of infections in Wuhan. On the other hand, infection cases of other prefectures appeared late, so the reported case data are more accurate. Based on the above considerations, we used the cumulative number of confirmed cases in mainland China (excluding Hubei Province) from January 24 to February 17 for model parameter fitting. We solved this nonlinear optimization using the *fmincon* function in MATLAB. A set of possible values of the parameters were obtained, which are shown in the Table A2. Then, we simulated the early spread of COVID-19 in mainland China used these possible parameters. Figure 4b shows the officially released data of confirmed cases and the epidemic development curve predicted by the model.

## 4. Discussion

In this study, considering the availability and accuracy of airline operation data and the large sample size of the mobile device location big data, we designed an algorithm model to estimate the inter-prefecture human travel flow in mainland China. The data required for the model are mainly the proportion of migration and the proportion of transportation modes. In other areas where such data are available, the human mobility pattern can also be estimated by this model. Our estimated human mobility pattern in mainland China shows that individuals’ travel distances are subject to long-tailed distribution, which is consistent with the general conclusions of human mobility in other studies. In addition, individuals in China are more inclined to travel between prefectures of the same province. Compared to traditional statistical survey data, mobile device location big data can provide detailed and dynamic personnel location changes in real time, and the large-scale human mobility patterns estimated using mobile device location big data can better reveal the characteristics of human social activities.

The use value of travel flow data estimated by our model is demonstrated in the work reproducing the spread of COVID-19. The cumulative number of confirmed cases in each prefecture is highly correlated with the estimated number of travelers from Wuhan. Moreover, in the established spatial spread mechanism model, the predicted curve fits the real confirmed case data well. However, there are several limitations in epidemic simulation modeling. First, it should be pointed out that we did not consider the stochasticity factor in the simulation and we simply established a deterministic epidemic model. Second, we fitted the epidemic parameters via using just one time series data. When solving nonlinear programming problems, what we want to do most is to calculate a global minimizer. However, this is very difficult, and finding a local minimizer through numerical algorithms is the best attempt we can do. Thus, it is difficult to guarantee that a unique set of values of parameters that gives the best fit can be obtained. Therefore, the uncertainty of the solution will cause that we cannot guarantee that the obtained parameters fit reality because the values of the parameters have certain realistic epidemiological significance. Our simulated epidemiological transmission may only guarantee that the number of confirmed cases fit reality, while the dynamics of other compartments may be different from the real scenario. We hope that the public health department will release more anonymized cases data, and that researchers with these data will carry out more in-depth studies on the epidemiological parameters.

In addition, considering the complexity of establishing a stochastic SEIR model on 337 prefectures, we simply divided mainland China into three subpopulations in this study. Follow-up work can establish metapopulation models for all prefectures to obtain higher resolution simulation results. Furthermore, researchers can obtain the proportional data of transportation modes among all prefectures in mainland China, based on which number of inter-prefecture travelers with different transportation modes can be calculated. Therefore, researchers can model and study the spread of infectious diseases with different means of transportation.

## Data Availability

Part of the results data presented in this study are available in Supplementary Material. The other data supporting the findings of this study are available on publicly accessible websites within the bibliography.

## Supplementary Materials

The following are available online at https://www.mdpi.com/article/10.3390/healthcare9091224/s1, Figure S1: Pseudocode of the algorithm used for generating bipartite network and estimating total arrival and total departure population of each administrative.

## Appendix A

The administrative division system of China can be described as “Province–Prefecture–County”. Specifically, as for the first-level division, the Constitution clearly states that China is divided into 23 provinces, five autonomous regions, four centrally-administered municipalities (i.e., Beijing, Shanghai, Tianjin, Chongqing), and two special administrative regions (i.e., Hong Kong, Macau). These 34 regions are provincial-level administrative regions. The subdivision of the provincial-level administrative region is the prefecture. The subdivision of the prefecture is the county. In this study, we estimated human mobility networks composed of 333 prefectures and four municipalities (metropolises) in mainland China. In order to avoid confusion, four municipalities were treated as prefectures in this study.

## Appendix B

Table A1 shows the example of the migration proportion data obtained from the Baidu Map Migration Big Data Platform. For instance, one source/destination prefecture of Beijing is Langfang and the move type is ‘move in’, which indicates that there are individuals who move from Langfang to Beijing. One source/destination prefecture of Beijing is Baoding and the move type is ‘move out’, which indicates that there are individuals who move from Beijing to Baoding. The data lists 100 source prefectures and 100 destination prefectures for each prefecture every day, and it is sorted by the migration proportion.

**Table A1:** Example table of the migration proportion data.

Order Number	Prefecture Code	Prefecture Name	Source/Destination Prefecture	Proportion (%)	Date	Move Type
1	110000	Beijing	Langfang	13.74	2020-01-02	Move in
2	110000	Beijing	Baoding	7.83	2020-01-02	Move in
…						
100	110000	Beijing	Foshan	0.16	2020-01-02	Move in
1	110000	Beijing	Langfang	9.27	2020-01-02	Move out
2	110000	Beijing	Baoding	7.12	2020-01-02	Move out
…						
100	110000	Beijing	Mudanjiang	0.19	2020-01-02	Move out

## Appendix C

**Table A2:** Definition of the parameters and possible values obtained by solving optimization problem.

Parameters	Notation	Values	Source	Interpretations
probability of transmission	β	0.028964	CNLP	probability of transmission per effective contact
number of contacts	$c_{1}$	21.332	CNLP	number of effective contacts in 1st period
	$c_{2}$	7.3657	CNLP	number of effective contacts in 2nd period
proportion of isolation: $q\left(t\right)=\frac{1}{1+e^{a-b\cdot \left(t-23\right)}}$	a	3.6818	CNLP	coefficient of Sigmoid function
	b	0.63232	CNLP	coefficient of Sigmoid function
coefficient of difference	ν	0.25175	CNLP	coefficient of difference between E and I
rate of transformation between compartments	λ	0.071429	[44]	rate at which the quarantined uninfected were released
	σ	0.20000	[46]	rate at which the infected from E to I
	$\delta_{I1}$	0.073912	CNLP	rate at which person from I to C in 1st period
	$\delta_{I2}$	0.18210	CNLP	rate at which person from I to C in 2nd period
	$\delta_{q1}$	0.017459	CNLP	rate at which person from $E^{q}$ to C in 1st period
	$\delta_{q2}$	0.42601	CNLP	rate at which person from $E^{q}$ to C in 2nd period
	γ	0.095992	CNLP	rate at which the infected from I to R
proportion	κ	0.51317	CNLP	proportion of symptomatic infected individuals that can travel normally
	$Min\left(P_{0}\right)$	7.9 × 10^4^	CNLP	local minimum of error

## References

[1] Colizza V., Vespignani A. (2008). Epidemic modeling in metapopulation systems with heterogeneous coupling pattern: Theory and simulations. J. Theor. Biol..

[2] Einav L., Levin J. (2014). Economics in the age of big data. Science.

[3] Xu J., Li A., Li D., Liu Y., Du Y., Pei T., Ma T., Zhou C. (2017). Difference of urban development in China from the perspective of passenger transport around Spring Festival. Appl. Geogr..

[4] Wei Y., Song W., Xiu C., Zhao Z. (2018). The rich-club phenomenon of China’s population flow network during the country’s spring festival. Appl. Geogr..

[5] Cui C., Wu X., Liu L., Zhang W. (2020). The spatial-temporal dynamics of daily intercity mobility in the Yangtze River Delta: An analysis using big data. Habitat Int..

[6] Kraemer M.U., Sadilek A., Zhang Q., Marchal N.A., Tuli G., Cohn E.L., Hswen Y., Perkins T.A., Smith D.L., Reiner R.C. (2020). Mapping global variation in human mobility. Nat. Hum. Behav..

[7] Meslé M.M.I., Hall I.M., Christley R.M., Leach S., Read J.M. (2019). The use and reporting of airline passenger data for infectious disease modelling: A systematic review. Eurosurveillance.

[8] Wilder-Smith A. (2006). The severe acute respiratory syndrome: Impact on travel and tourism. Travel Med. Infect. Dis..

[9] Fraser C., Donnelly C.A., Cauchemez S., Hanage W.P., Van Kerkhove M.D., Hollingsworth T.D., Griffin J., Baggaley R.F., Jenkins H.E., Lyons E.J. (2009). Pandemic potential of a strain of influenza A (H1N1): Early findings. Science.

[10] Lopez L.F., Amaku M., Coutinho F.A.B., Quam M., Burattini M.N., Struchiner C.J., Wilder-Smith A., Massad E. (2016). Modeling importations and exportations of infectious diseases via travelers. Bull. Math. Biol..

[11] Quam M.B., Khan K., Sears J., Hu W., Rocklöv J., Wilder-Smith A. (2015). Estimating air travel–associated importations of dengue virus into Italy. J. Travel Med..

[12] Quam M.B., Wilder-Smith A. (2015). Importation index of dengue to determine the most probable origin of importation. J. Travel Med..

[13] Brockmann D., Helbing D. (2013). The hidden geometry of complex, network-driven contagion phenomena. Science.

[14] Ravenstein E.G. (1885). The laws of migration. J. Stat. Soc. Lond..

[15] Simini F., González M.C., Maritan A., Barabási A.-L. (2012). A universal model for mobility and migration patterns. Nature.

[16] Huang Z., Wu X., Garcia A.J., Fik T.J., Tatem A.J. (2013). An open-access modeled passenger flow matrix for the global air network in 2010. PLoS ONE.

[17] Mao L., Wu X., Huang Z., Tatem A.J. (2015). Modeling monthly flows of global air travel passengers: An open-access data resource. J. Transp. Geogr..

[18] Balcan D., Colizza V., Gonçalves B., Hu H., Ramasco J.J., Vespignani A. (2009). Multiscale mobility networks and the spatial spreading of infectious diseases. Proc. Natl. Acad. Sci. USA.

[19] Ajelli M., Gonçalves B., Balcan D., Colizza V., Hu H., Ramasco J.J., Merler S., Vespignani A. (2010). Comparing large-scale computational approaches to epidemic modeling: Agent-based versus structured metapopulation models. BMC Infect. Dis..

[20] Balcan D., Gonçalves B., Hu H., Ramasco J.J., Colizza V., Vespignani A. (2010). Modeling the spatial spread of infectious diseases: The GLobal Epidemic and Mobility computational model. J. Comput. Sci..

[21] Candia J., González M.C., Wang P., Schoenharl T., Madey G., Barabási A.-L. (2008). Uncovering individual and collective human dynamics from mobile phone records. J. Phys. A Math. Theor..

[22] Gonzalez M.C., Hidalgo C.A., Barabasi A.-L. (2008). Understanding individual human mobility patterns. Nature.

[23] Xiong C., Hu S., Yang M., Luo W., Zhang L. (2020). Mobile device data reveal the dynamics in a positive relationship between human mobility and COVID-19 infections. Proc. Natl. Acad. Sci. USA.

[24] Coronaviridae Study Group of the International Committee on Taxonomy of Viruses (2020). The species Severe acute respiratory syndrome-related coronavirus: Classifying 2019-nCoV and naming it SARS-CoV-2. Nat. Microbiol..

[25] Kraemer M.U., Yang C.-H., Gutierrez B., Wu C.-H., Klein B., Pigott D.M., Du Plessis L., Faria N.R., Li R., Hanage W.P. (2020). The effect of human mobility and control measures on the COVID-19 epidemic in China. Science.

[26] Badr H.S., Du H., Marshall M., Dong E., Squire M.M., Gardner L.M. (2020). Association between mobility patterns and COVID-19 transmission in the USA: A mathematical modelling study. Lancet Infect. Dis..

[27] Cartenì A., Di Francesco L., Martino M. (2020). How mobility habits influenced the spread of the COVID-19 pandemic: Results from the Italian case study. Sci. Total Environ..

[28] Lai S., Ruktanonchai N.W., Zhou L., Prosper O., Luo W., Floyd J.R., Wesolowski A., Santillana M., Zhang C., Du X. (2020). Effect of non-pharmaceutical interventions to contain COVID-19 in China. Nature.

[29] Chang S., Pierson E., Koh P.W., Gerardin J., Redbird B., Grusky D., Leskovec J. (2021). Mobility network models of COVID-19 explain inequities and inform reopening. Nature.

[30] Ruktanonchai N.W., Floyd J., Lai S., Ruktanonchai C.W., Sadilek A., Rente-Lourenco P., Ben X., Carioli A., Gwinn J., Steele J. (2020). Assessing the impact of coordinated COVID-19 exit strategies across Europe. Science.

[31] Pepe E., Bajardi P., Gauvin L., Privitera F., Lake B., Cattuto C., Tizzoni M. (2020). COVID-19 outbreak response: A first assessment of mobility changes in Italy following national lockdown. Sci. Data.

[32] Pan Y., Darzi A., Kabiri A., Zhao G., Luo W., Xiong C., Zhang L. (2020). Quantifying human mobility behaviour changes during the COVID-19 outbreak in the United States. Sci. Rep..

[33] Kang Y., Gao S., Liang Y., Li M., Rao J., Kruse J. (2020). Multiscale dynamic human mobility flow dataset in the US during the COVID-19 epidemic. Sci. Data.

[34] Baidu Map Migration Big Data Platform. http://qianxi.baidu.com/.

[35] Tencent location Big Data. https://heat.qq.com/.

[36] VariFlight Big Data. https://data.variflight.com/.

[37] National Health Commission of the People’s Republic of China. http://www.nhc.gov.cn/xcs/yqtb/list_gzbd.shtml.

[38] Mahikul W., Chotsiri P., Ploddi K., Pan-ngum W. (2021). Evaluating the Impact of Intervention Strategies on the First Wave and Predicting the Second Wave of COVID-19 in Thailand: A Mathematical Modeling Study. Biology.

[39] Wölfel R., Corman V.M., Guggemos W., Seilmaier M., Zange S., Müller M.A., Niemeyer D., Jones T.C., Vollmar P., Rothe C. (2020). Virological assessment of hospitalized patients with COVID-2019. Nature.

[40] Pan A., Liu L., Wang C., Guo H., Hao X., Wang Q., Huang J., He N., Yu H., Lin X. (2020). Association of Public Health Interventions With the Epidemiology of the COVID-19 Outbreak in Wuhan, China. JAMA J. Am. Med. Assoc..

[41] Nishiura H., Kobayashi T., Suzuki A., Jung S.M., Miyama T. (2020). Estimation of the asymptomatic ratio of novel coronavirus infections (COVID-19). Int. J. Infect. Dis..

[42] Protocol on Prevention and Control of COVID-19 (Edition 6). http://en.nhc.gov.cn/2020-03/29/c_78468.htm.

[43] Zhang J., Litvinova M., Liang Y., Wang Y., Yu H. (2020). Changes in contact patterns shape the dynamics of the COVID-19 outbreak in China. Science.

[44] Chong E., Zak S.H. (2013). An Introduction to Optimization.

[45] Rosvall M., Bergstrom C.T. (2008). Maps of Random Walks on Complex Networks Reveal Community Structure. Proc. Natl. Acad. Sci. USA.

[46] Li Q., Guan X., Wu P., Wang X., Zhou L., Tong Y., Ren R., Leung K.S.M., Lau E.H.Y., Wong J.Y. (2020). Early Transmission Dynamics in Wuhan, China, of Novel Coronavirus–Infected Pneumonia. N. Engl. J. Med..

