# Supplementary material for: Estimation of Human Mobility Patterns for Forecasting the Early Spread of Disease
Source: Healthcare (Basel). 2021 Sep 16;9(9):1224. doi: 10.3390/healthcare9091224 (PMC8468459; doi:10.3390/healthcare9091224)
Supplement: Supplementary file 1 [file healthcare-09-01224-s001.zip › Supplementary Material/Figure S1/pseudocode.pdf]

Data: *Baidu Map Migration Big Data*, *Tencent Location Big Data*, airline operation data

Result: Total arrival and total departure population of each administrative region

- Convert *Baidu Map Migration Big Data* into the structural information of the star network
- For each region i , generate a node of type *departure*: i^{out}, and a node of type *arrival*: i^{in}
- **for** each region i

  for each neighbor node j of i in the *departure* star network G_i^{out}

  while i in the *arrival* star network G_j^{in}

  let p_{ij}^{out} denote the weight of edge e_{ij}^{out} in network G_i^{out}

  let p_{ij}^{in} denote the weight of edge e_{ij}^{in} in network G_j^{in}

  connect an edge between node i^{out} and node j^{in}, weight of the edge is set to p_{ij}^{out}/p_{ij}^{in}

  end for

  end for
- Select a node, such as i^{out}, estimate its status information N_i^{out}, which is used as the initial input information, then put the node into the set of nodes to be visited S_1
- **for** each node v in the set S_1

  let the current node be v

  remove v from the set S_1, and put it into the set of nodes have been visited S_2

  for each neighbor node n of node v

  while the node n is neither in the set S_1 nor in the set S_2

  put the node n into the set S_1

  while the status information of the node n has not been estimated

  while the type of v is *departure*

  set estimated value of the status information of the node n to $N_n^{in}=N_v^{out}*p_{vn}^{out}/p_{vn}^{in}$

  while the type of v is *arrival*

  set estimated value of the status information of the node n to $N_n^{out}=N_v^{in}*p_{nv}^{in}/p_{nv}^{out}$

  end for

  end for
